# Circulating cell-free DNA and its integrity as a prognostic marker for breast cancer

**DOI:** 10.1186/s40064-015-1071-y

**Published:** 2015-06-17

**Authors:** Sobuhi Iqbal, Sreenivas Vishnubhatla, Vinod Raina, Surabhi Sharma, Ajay Gogia, Suryanarayana S V Deo, Sandeep Mathur, Nutan Kumar Shukla

**Affiliations:** Department of Medical Oncology, Dr. B.R.A. Institute Rotary Cancer Hospital, All India Institute of Medical Sciences, New Delhi, India; Department of Biostatistics, All India Institute of Medical Sciences, New Delhi, India; Department of Medical Oncology, Hematology and Stem Cell Transplantation, Fortis Memorial Research Institute, Sector 44, Gurgoan, India; Department of Surgical Oncology, Dr. B.R.A. Institute Rotary Cancer Hospital, All India Institute of Medical Sciences, New Delhi, India; Department of Pathology, All India Institute of Medical Sciences, New Delhi, India

**Keywords:** Circulating cell-free DNA, DNA integrity, Breast cancer, Surgery

## Abstract

The aim of our study was to look for alternative predictive biomarkers for breast cancer management in limited resource setup. A comprehensive analysis of circulating cell-free DNA (CCFD) in serum at baseline was performed to assess its prognostic potential. Quantitative polymerase chain reaction (qPCR) of ALU sequences using ALU115 and ALU247 primers was carried out in patients (N: baseline 148, postoperative 47) and 51 healthy controls. Mean serum DNA integrity, levels of ALU 247 and levels of ALU 115 were significantly higher in patients than in healthy females. No significant differences were observed in the levels ALU 247 and ALU 115 between stage IV and earlier stages of the disease. The DNA integrity was significantly higher in stage IV than earlier stages. A significant decrease in DNA integrity was observed after surgery (pre: 0.55 ± 0.23 vs post: 0.43 ± 0.30; P = 0.002) while no such change could be observed for ALU 247 and ALU 115. Baseline DNA integrity was significantly higher in relapsed patients than in patients who were free of disease (P = 0.005). Higher baseline DNA integrity was also indicated, though statistically not significant, in patients who died (P = 0.14). In contrast, ALU 247 and ALU 115 levels were decreased in died patients as compared to survivors (24.8 ± 34.80 vs 73.5 ± 170.83, P = 0.02 for ALU 247 and 41.0 ± 47.99 vs 159.5 ± 299.54, P = 0.005 for ALU 115). Baseline levels of ALU 115 and ALU 247 were lower in relapsed patients, though statistically not significant. In univariate analysis, the only clinic-pathological parameter associated with disease prognosis was tumor size. The hazards of 5-year overall mortality was 3.60 (95 % CI: 1.03 12.53, P = 0.03) among patients with lower baseline serum levels of CCFD (ALU 247 < 21 and ALU 115 < 41). Similarly the 4 year hazards for recurrence was 2.30 (95 % CI: 0.96 5.52, P = 0.05) among patients with higher DNA integrity. Baseline serum levels of CCFD and its integrity were found to be potential prognostic biomarkers in patients of primary breast cancer at our centre.

**Electronic supplementary material:**

The online version of this article (doi:10.1186/s40064-015-1071-y) contains supplementary material, which is available to authorized users.

## Background

Breast cancer is the most common cancer in women worldwide, including India, and is the leading cause of death from cancer among women globally (Ferlay et al. 2015). It is estimated that 145,000 new cases are diagnosed and 70,000 deaths occur annually in India (Ferlay et al. 2015). The average age at diagnosis is 43–46 years in Indian women, about 10 years lower than the age in the western world (Saxena et al. 2005) and in Asian population, increase in incidence of breast cancer is associated with increased mortality (Ferlay et al. 2015). Improvement in treatment strategies for breast cancer has resulted in longer survival of patients, with a need for suitable and optimal follow-up, mainly during the first 3 years, which is associated with locoregional and distant recurrences (Clarke and Martinez 1992). At present there is no improvement in the surveillance methods used to detect the recurrences and the serological markers available have not improved the rate of recurrences (Duffy 2006). Hence, there is a need for developing new biomarkers which can be used as valuable tools in identifying high risk patients, to predict disease prognosis and thus have an impact in patient management. In view of the infrastructural limitations and affordability of the facilities etc. (Dinshaw et al. 2005; Okonkwo et al. 2008), alternative approaches to breast cancer monitoring are clearly needed.

Presence of circulating cell-free DNA (CCFD) in blood, though reported in 1948 (Mandel and Metais 1948), was rediscovered after 30 years in autoimmune disorders (Tan et al. 1966) and in cancer (Leon et al. 1977). Its presence in blood is studied by quantification, mutation, methylation as well as microsatellite alteration (Jung et al. 2010). The higher levels of cell-free DNA are found in serum or plasma of cancer patients than in healthy controls (Sunami et al. 2008; Stötzer et al. 2014). In healthy individuals, the main source of cell free DNA in circulating blood is through apoptosis, whereas in cancer patients it results from both apoptosis as well as by necrosis (Jahr et al. 2001). The major portion of CCFD in cancer patients is of apoptotic origin i.e. shorter fragments, therefore elevated levels of longer fragments of DNA in blood has been reported to be a good marker for the presence of malignant tumor DNA (Jahr et al. 2001; Umetani et al. 2006c; Diehl et al. 2008). Based on this logic, DNA integrity, the ratio of longer to shorter DNA fragments, has been assessed for its diagnostic and prognostic potential in cancer patients (Wang et al. 2003; Umetani et al. 2006a, c; Holdenrieder et al. 2008; El-Shazly et al. 2010). DNA integrity has been reported to be significantly higher in patients with metastatic breast cancer as compared to patients with locally confined breast cancer and benign controls (Stötzer et al. 2014). It was found to predict tumor progression, regional LN metastases in primary breast cancer patients (Umetani et al. 2006a) and shorter disease free interval in patients with nasopharyngeal cancer (Chan et al. 2008). However, recent evidence suggests that it has no role in predicting the response to therapy in breast cancer patients who received neoadjuvant chemotherapy (Lehner et al. 2013). The diverse results in literature regarding the predictive ability of CCFD in primary breast cancer are on account of the fact that most of studies have evaluated either the absolute levels of CCFD or DNA integrity (Jung et al. 2010).

In the present study, the aim was to assess the utility of CCFD as an alternative prognostic tool in the limited resource setup. We studied the baseline serum levels of absolute DNA, longer fragments of DNA as well as its integrity in primary breast cancer patients and comprehensively evaluated the predictive ability of each biomarker with overall survival (OS) as well as disease free survival (DFS) of these patients.

## Methods

### Patient selection

We included newly diagnosed primary breast cancers attending the outpatient clinic of the department of Medical Oncology at Institute Rotary Cancer Hospital, All India Institute of Medical Sciences, New Delhi, India during April 2009 to February 2012. A written informed consent was obtained from all participants and the Institute’s Ethics Committee approved the study.

### Inclusion criteria

1. Pathological diagnosis of primary breast cancer.
2. Treatment naïve.
3. Age ≥18 years.

### Samples and clinico-pathological information

Venous blood samples (10 ml in a serum separator tube) were collected from cases with American Joint Committee on Cancer (AJCC) stage I to IV of primary breast cancers. A subset of patients was sampled 1 month after modified radical mastectomy. In addition, blood samples were also collected from healthy female volunteers. All samples were processed immediately.

### Serum preparation for direct quantitative PCR (qPCR)

The serum was separated by centrifugation (1,000×*g*, 15 min) and was passed through a 13-mm serum filter (Millipore) to remove any contaminating cells, divided into aliquots in clean polypropylene tubes and stored at −80°C until processing. Serum preparation for qPCR was performed as described by Umetani et al. (2006a) Briefly, serum proteins which might hinder the qPCR results by binding to template DNA or DNA polymerase were deactivated by mixing 20 µL of each serum sample with 20 µL of a preparation buffer that contained 2.5% of tween 20, 50 mmol/L Tris, and 1 mmol/L EDTA. This mixture was digested with proteinase K (20 µg) solution for 50 min (Promega) at 56°C, followed by 5 min of heat deactivation and insolubilization for 10 min at 95°C. After subsequent centrifugation of 10,000×*g* for 5 min, 0.2 µL of supernatant was used as a template for each qPCR reaction.

### Quantitative PCR of ALU repeats

Quantitative PCR was performed as described previously (Umetani et al. 2006a). Two primer sets amplifying ALU sequences were used to quantify the levels of a 115 bp DNA amplicon (ALU115 amplifying both short and long DNA fragments), and a 247 bp amplicon (ALU247, amplifying only large DNA fragments), the ALU115 primers were forward: 5_-CCTGAGGTCAGGAGTTCGAG-3′ and reverse: 5′-CCCGAGTAGCTGGGATTACA-3′; and ALU247 primers were forward: 5′-GTGGCTCACGCCTGTAATC-3′ and reverse: 5′-CAGGCTGGAGTGCAGTGG-3′. ALU115-qPCR values represent the total amount of CCFD in serum, whereas ALU247-qPCR values represent amounts of DNA in serum released from non-apoptotic cells. DNA integrity was calculated as the ratio of qPCR results (ALU247-qPCR/ALU115-qPCR). As the annealing sites of ALU115 are within the ALU247 annealing sites, thus the ratio of ALU247 to ALU115 is termed as DNA integrity. It characterizes the fragmentation pattern of CCFD (i.e. the DNA integrity is 1 if template DNA is not truncated and 0 if DNA is completely truncated to fragments smaller than 247 bp). Quantitative PCR was carried out in duplicates on LightCycler2 (ROCHE). Each 20 μl reaction consisted of 1 × LightCycler DNA Master SYBR Green I (ROCHE), 400 nM forward and reverse primers, 5 mM MgCl_2_ and 0.2 μl of DNA sample. PCR amplification was carried out according to manufacturer’s instruction which was 95°C for 30 s, followed by 45 cycles at 95°C for 0 s and 57/55°C for 10 s and 72 for 5 s followed by melting curve analysis to confirm specificity of the PCR products. Each run included fivefold dilutions of an external standard (healthy leukocyte DNA) and negative control (without template).

### Clinical follow-up

Starting from the completion of treatment, patients were followed up every 3 months during the first year, every 6 months during the second year, and then yearly until relapse with clinical, biochemical, and radiological examinations. All the patients received multimodality treatment in the form of surgery (for stages I–III), chemotherapy, radiotherapy and hormonal ± targeted therapy whenever indicated. Overall survival (OS) was defined as the period from date of diagnosis to death and Disease Free Survival (DFS) was defined as the period from end of treatment to relapse.

### Statistical analysis

Data was expressed as mean ± standard deviation (SD). Differences between groups were assessed by using Student’s *t* test. Differences in study parameters between pre and post-operative groups were assessed using paired *t*-test. Log transformed values were used for analyses. A *P* value ≤0.05 was considered as significant. OS and DFS were assessed by Kaplan–Meier survival analysis. The association of CCFD and DNA integrity with survival was evaluated by Cox proportional hazard regression model. All statistical analysis was done using Stata 12.1.

## Results

### Patient characteristics

One hundred forty-eight patients and 51 healthy female volunteers were enrolled. Repeat serum samples from 47 post operative patients were studied. Mean age of the patients was 48.2 ± 10.79 years and that of controls was 42.3 ± 11.4 years. Average tumor size was 4.6 ± 2.70 cm among patients undergoing surgery. Patient characteristics are summarized in Additional file 1: Table S1. As can be seen most of the cases had ductal carcinoma and about a fifth of them were triple negative.

### Circulating cell-free DNA levels—ALU247 (qPCR values of longer DNA fragments)

Primary breast cancers showed significantly (P < 0.001) higher levels of ALU247 as compared to healthy females. Levels of ALU247 were highest in stages I–III (63.8 ± 154.77), least in healthy controls (11.4 ± 9.01) and patients of stage IV showed in between (48.4 ± 63.73). The difference in the levels between stage IV and earlier stages was not statistically significant. Levels of ALU247 were significantly elevated in stages I–III and also in Stage IV as compared healthy controls (Additional file 1: Table S2).

The levels of ALU247 did not alter significantly after the surgery (pre: 29.9 ± 49.38 vs post: 24.7 ± 23.49 pg/μL, P = 0.15, N = 47) among cases of stages I-III. Postoperative ALU247 levels of these cases were still higher than the levels in healthy controls (24.7 ± 23.49 vs 11.4 ± 9.01, P = 0.06).

The mean baseline levels of ALU247 were elevated in patients of stages I–III without relapse as compared to patients with relapse, though not statistically significant (Additional file 1: Table S3), but significantly elevated in survivors (73.5 ± 170.83) than in patients who died (24.8 ± 34.80). In contrast, no statistically significant differences were observed between died and alive patients of stage IV (Additional file 1: Table S3).

### ALU115 (qPCR values of both shorter and longer DNA fragments)

The levels of ALU115 were significantly higher in patients than in healthy controls (136.3 ± 272.50 vs 39.1 ± 22.96, P = 0.05). The levels were similar in cases of stages I–III and stage IV.

Post surgical levels of ALU115 though elevated, were not significantly different from the pre-surgical levels (126.1 ± 249.22 vs 67.7 ± 123.42 pg/μL, P = 0.15, N = 47) among cases of stages I-III (Additional file 1: Table S2). The post-surgical levels of ALU115 were significantly higher than that of healthy controls (126.1 ± 249.22 vs 39.1 ± 22.96, P = 0.03).

The relapsed and died patients showed lower levels of ALU115 as compared to relapse free and alive patients respectively. However, the reduced levels among relapsed patients were not significant from the relapse free patients. Patients of stage IV did not show any difference in the levels of ALU115 between died and alive patients (Additional file 1: Table S3).

### DNA integrity (ALU247/ALU115)

The mean serum DNA integrity was significantly elevated in patients than in healthy females (Additional file 1: Table S2). Further, an increasing trend of DNA integrity was observed from healthy controls (0.35 ± 0.27) to breast cancer stages of I–III (0.53 ± 0.23) to stage IV cases (0.60 ± 0.26).

DNA integrity levels came down significantly after surgery among cases of stage I–III (0.55 ± 0.23 vs 0.43 ± 0.30; P = 0.002). The post-operative levels of DNA integrity in these patients was comparable to that of healthy females (0.43 ± 0.30 vs 0.35 ± 0.27, P = 0.94).

The baseline DNA integrity was significantly higher in relapsed patients than patients without relapse (0.65 ± 0.23 vs 0.49 ± 0.21, P = 0.005). No significant differences were observed between died and alive patients, among both stages I–III and stage IV (Additional file 1: Table S3).

The analysis of the association of various clinico-pathological factors with overall survival and disease free survival indicated that only the size of the tumor (≥4.0 cm) to be associated with poorer overall survival (HR: 3.56, 95% CI 1.02–12.02, P = 0.03), Additional file 1: Table S4. When each patient was categorized as high or low based on the median levels of each biomarker among alive patients, only ALU115 showed marginally significant association with OS. However, the combination of ALU247 and ALU115 showed significant association (Additional file 1: Table S4). Compared to those cases of stages I-III with ALU247 ≥ 21 and ALU115 ≥ 41 pg/µL, cases with either one below the respective cutoff values had a HR of 3.6 (95% CI 1.03–12.53), Additional file 1: Table S4. Cases with tumor size ≥ 4.0 cm and ALU247 < 21 and/or ALU115 < 41 had a hazards of 6.30 (95% CI: 2.05–19.36, P = 0.001).

The size of the tumor and DNA integrity showed significant association with disease free survival (Additional file 1: Table S4). Patients with a tumor size ≥4.0 cm had a hazards of 3.4 compared to those with a tumor size of <4 cm. Those patients whose DNA integrity was ≥0.48 had higher hazards of 2.30 (Additional file 1: Table S4). When both the size of tumor and the DNA integrity were assessed together, both showed a significant association with DFS, HR for size of tumor was 3.91 (95% CI 1.46–10.48, P = 0.01); and HR for DNA integrity was 2.85 (95 % CI 1.18–6.86, P = 0.02).

## Discussion

This prospective observational study included a cohort of early and locally advanced breast cancers. The levels of CCFD in serum were evaluated by qPCR of ALU sequences, which are 300 base pair (bp) interspersed repeat elements in the human genome, having a copy number of approximately 1 million copies per genome (Deininger 2011). Serum was used for studying the CCFD (ALU247 & ALU115) levels, as it is believed to be better source of CCFD than plasma (Umetani et al. 2006b). The amount of DNA found in the serum is high with low levels of contaminating DNA released from blood cells when processed within 8 h after collection (Umetani et al. 2006a).

The clearance of apoptotic and necrotic debris by infiltrating phagocytes is hampered during tumor growth, thus resulting in accumulation of cellular debris and its secretion into the circulation (Diaz and Bardelli 2014). In addition, the active secretion of apoptotic and necrotic DNA in circulation has also been indicated in the increase of CCFD levels in blood (Jahr et al. 2001). A number of studies have highlighted the presence of significantly elevated levels of CCFD in plasma as well as in the serum of patients suffering from breast, colorectal, lung and other cancers (Umetani et al. 2006c; Holdenrieder et al. 2008; Yoon et al. 2009; Agostini et al. 2012). In the present study, we too observed significantly higher levels of longer DNA fragments (ALU247), absolute DNA (ALU115) as well as higher DNA integrity in breast cancer patients than in healthy controls. We observed that the DNA integrity was indicative of tumor progression which is supported by the study of association between DNA integrity and tumor progression in breast cancer patients (Umetani et al. 2006a).

The elevated levels of CCFD levels due to trauma reach normal levels within 2 h after traumatic injury and the half-life of CCFD is approximately 16 min (Lo et al. 1999; Lam et al. 2003), so following surgery, a gap of 4–5 weeks was given prior to next sampling. Some studies have reported that the CCFD levels decrease following surgery (Catarino et al. 2008), whereas others report that there is persistence of tumor DNA after surgery in the plasma of breast cancer patients (Silva et al. 2002a). We did not observe any significant decrease in the levels of ALU247; however DNA integrity decreased significantly after surgery which indicates more of apoptotic DNA in circulation. The higher levels of ALU115 observed in patients after surgery might be due to the increase in apoptosis associated with wound healing (Greenhalgh 1998; Wu and Chen 2014). The persistence of longer fragments of DNA after excision of tumor indicates that its presence in circulation might be associated with some other body mechanism.

The prognostic value of CCFD has only been explored in a small number of studies which report that the decreasing CCFD levels are associated with early treatment response, e.g. in lung cancer (Gautschi et al. 2004), rectal cancer during neo-adjuvant chemo-radiation therapy (Zitt et al. 2008) and in breast cancer (Lehner et al. 2013). In contrast, we did not observe any association of post-operative levels of ALU247, ALU115 and DNA integrity with either OS or DFS (data not shown). However, our pre-therapeutic serum levels of CCFD demonstrate that percentage of survival was better in patients with higher levels of ALU247 as well as ALU115. These results are in line with study by Gal et al., which show that the breast cancer patients with higher concentration of serum DNA had better overall survival, which they linked with a better host response to the tumor so improved survival rates (Gal et al. 2004).

The persistence of increased DNA integrity observed after the treatment in nasopharyngeal tumors indicate worse prognosis than patients with reduced DNA integrity (Chan et al. 2008). However, recently Lehner et al., reported that DNA integrity was not informative of therapy outcome in breast cancer patients (Lehner et al. 2013). In contrast in the present study, we observed that the baseline DNA integrity was associated with DFS i.e. patients with higher DNA integrity had poorer DFS. These results are consistent with the study of Silva et al. who reported that cell-free tumor DNA at diagnosis in plasma of breast cancer patients is associated with DFS (Silva et al. 2002b).

In the present study, we observed that the size was the only clinic-pathological marker which was associated with both the OS and DFS of the patients, which is a well documented prognostic factor of breast cancer (Carter et al. 1989). One study has reported a borderline significance between the plasma tumor DNA and DFS in multivariate analysis with tumor size, lymph node and tumor stage (Silva et al. 2002b). Our multivariable analysis showed that the tumor size and CCFD (ALU247, ALU 115) were significant prognostic factors associated with 5-year OS, while tumor size in association with DNA integrity significantly predicted 4-year DFS of primary breast cancer. So tumor size in association with CCFD might be helpful in identification of high risk patients and their early management.

The varied results regarding the predictive and prognostic utility of CCFD reported in the literature are because of the lack of standardized procedures for its quantification. In order to validate the clinical utility of CCFD, there is a need to harmonize the techniques involved in its quantification and also to include well-powered sample size in study.

In conclusion, our results show that the baseline serum levels of ALU247, ALU115 and DNA integrity are higher in patients than in healthy controls. An interesting correlation was observed between the baseline levels of ALU247, ALU115, DNA integrity and disease prognosis in patients (stages I–III). The levels of CCFD (ALU247, ALU115) were predictive of 5-year survival whereas the DNA integrity was indicative of 4-year DFS. Further studies are required to examine the association of levels of CCFD with host immune responses and its implication in the prognosis of breast cancer. The large standard deviation of these markers points out that there may be many other factors associated with them which need to be explored.

## Additional file

Additional file 1: In the supplemental material section the clinicopathological parameters, levels of CCFD & its integrity as well as their association with OS & DFS of patients are presented.

## Abbreviations

AJCC: American Joint Committee on Cancer
bp: base pairs
CCFD: circulating cell-free DNA
CI: confidence of interval
ER: estrogen receptor
PR: progesterone receptor
HER2: human epidermal growth factor receptor 2
TNBC: triple negative breast cancer
LN: lymph node
LVI: lympho vascular invasion
mins: minutes
MRM: modified radical mastectomy
PCR: polymerase chain reaction
PR: progesterone receptor
qPCR: quantitative PCR
SD: standard deviation
HR: hazard ratio
OS: overall survival
DFS: disease free survival
vs: versus
yrs: years.
