# Supplementary material for: Circulating cell-free DNA and its integrity as a prognostic marker for breast cancer
Source: Springerplus. 2015 Jun 17;4:265. doi: 10.1186/s40064-015-1071-y (PMC4469592; doi:10.1186/s40064-015-1071-y)
Supplement: Additional file 1: — In the supplemental material section the clinicopathological parameters, levels of CCFD & its integrity as well as their association with OS & DFS of patients are presented. [file 40064_2015_1071_MOESM1_ESM.docx]

**Table S1. Clinicopathologic characteristics of primary breast cancer cases**

| Characteristic | Frequency (%)  (N = 148) |
| --- | --- |
| Age (yrs) Mean ± SD | 48.2 ±10.79 |
| Duration of lump (months)  Mean ± SD  Median (IQR) | 8.8 ± 16.24  6 (2 - 12) |
| Localization  Right  Left | 61 (41.2)  87 (58.8) |
| Stage  I  II  III  IV | 4 (3)  39 (26)  43 (29)  62 (42) |
| Histology  Invasive Ductal  Invasive Lobular | 147 (99)  1 (1) |
| Hormone Receptor status  ER+ & PR+  ER- & PR–  One +, one -  Her2 +  Her2 –  TNBC | 53 (38)  74 (53)  12 (9)  82 (61)  53 (39)  28 (21) |

**Table S2. Baseline levels of CCFD and its integrity in healthy controls and primary breast cancer patients**

| Characteristic | Group | | | | P | | | |
| --- | --- | --- | --- | --- | --- | --- | --- | --- |
|  | Control  (N = 51) | Primary Breast cancer | | |  |  |  |  |
|  |  | Stage I, II & III  (N = 86) | Stage IV  (N = 62) | All cases  (N = 148) |  |  |  |  |
|  | 1 | 2 | 3 | 4 | 1 *vs* 2 | 1 *vs* 3 | 2 *vs* 3 | 1 *vs* 4 |
| ALU 247 (pg/µl)  Mean ± S.D | 11.4 ± 9.01 | 63.8 ± 154.77 | 48.4 ± 63.73 | 57.4 ± 124.88 | < 0.001 | < 0.001 | > 0.99 | < 0.001 |
| ALU 115 (pg/µl)  Mean ± S.D | 39.1 ± 22.96 | 136.1 ± 272.89 | 136.5 ± 274.18 | 136.3 ± 272.50 | 0.20 | 0.33 | > 0.99 | 0.05 |
| DNA Integrity  Mean ± S.D | 0.35 ± 0.27 | 0.53 ± 0.23 | 0.60 ± 0.26 | 0.56 ± 0.24 | < 0.001 | < 0.001 | 0.05 | < 0.001 |

**Table S3: Baseline levels of CCFD and integrity in association with outcome of primary breast cancer**

| Characteristic | Stage I, II & III | | | | | | Stage IV | | |
| --- | --- | --- | --- | --- | --- | --- | --- | --- | --- |
|  | No relapse  N = 61 | Relapse  N = 25 | P | Alive  N = 69 | Died  N = 17 | P | Alive  N = 30 | Died  N = 32 | P |
| ALU 247 (pg/µl)  Mean ± S.D | 74.9 ± 181.28 | 36.8 ± 40.12 | 0.46 | 73.5 ± 170.83 | 24.8 ± 34.80 | 0.02 | 40.9 ± 45.94 | 55.5 ± 76.88 | 0.80 |
| ALU 115 (pg/µl)  Mean ± S.D | 165.9 ± 317.01 | 63.2 ± 68.61 | 0.10 | 159.5 ± 299.54 | 41.0 ± 47.99 | 0.005 | 117.3 ± 191.81 | 154.5 ± 335.87 | 0.89 |
| DNA Integrity  Mean ± S.D | 0.49 ± 0.21 | 0.65 ± 0.23 | 0.005 | 0.51 ± 0.22 | 0.62 ± 0.25 | 0.14 | 0.60 ± 0.27 | 0.61 ± 0.26 | 0.85 |

**Table S4. Associations of study characteristics with overall and disease free survival of breast cancer patients (stages I-III)**

| **Characteristic** | **5 year Overall Survival** | | | | **4 Year Disease Free Survival** | | | | |
| --- | --- | --- | --- | --- | --- | --- | --- | --- | --- |
|  | N | %  Survival | HR (95% CI) | P | N | | %  Survival | HR (95% CI) | P |
| ER Positive  Negative | 36  48 | 82.7  76.6 | 1.00  1.39 (0.51 3.84) | 0.52 | 36  48 | | 65.7  65.6 | 1.13 (0.51 2.50) | 0.75 |
| PR Positive  Negative | 36  48 | 86.1  73.4 | 1.00  1.95 (0.68 5.61) | 0.20 | 36  48 | | 69.1  63.1 | 1.38 (0.62 3.06) | 0.43 |
| Her2 Positive  Negative | 48  33 | 77.8  83.8 | 1.00  0.75 (0.26 2.20) | 0.60 | 48  33 | | 72.7  55.3 | 1.76 (0.80 2.86) | 0.15 |
| TNBC Yes  No | 17  64 | 79.4  80.2 | 1.00  0.93 (0.26 3.28) | 0.90 | 17  64 | | 51.33  69.1 | 0.51 (0.21 1.22) | 0.13 |
| LN Negative  Positive | 30  54 | 83.3  78.8 | 1.00  1.27 (0.43 3.72) | 0.66 | 30  54 | | 77.3  59.9 | 2.11(0.84 5.29) | 0.10 |
| LVI Yes  No | 35  36 | 77.0  88.9 | 1.00  0.49 (0.14 1.67) | 0.24 | 35  36 | | 72.0  72.6 | 0.81(0.31 2.12) | 0.68 |
| Size < 4.0  ≥ 4.0 | 33  52 | 90.7  70.0 | 1.00  3.56 (1.02 12.02) | 0.03 | 33  52 | | 84.0  53.6 | 3.36 (1.26 8.96) | 0.01 |
| Menopause Yes  No | 42  44 | 77.8  79.3 | 1.00  0.94 (0.36 2.44) | 0.90 | 42  44 | | 69.7  64.7 | 0.97 (0.47 1.99) | 0.94 |
| ALU 247 ≥ 21 pg/µL  < 21 pg/µL | 43  43 | 84.5  73.3 | 1.00  1.82 (0.67 4.92) | 0.23 | 43  43 | | 64.9  69.4 | 0.98 (0.45 2.15) | 0.96 |
| ALU 115 ≥ 41 pg/µL  < 41 pg/µL | 39  47 | 88.7  71.2 | 1.00  2.68 (0.87 8.23) | 0.07 | ≥ 39 pg/µL (N = 43)  < 39 pg/µL (N = 43) | | 64.6  69.4 | 0.95 (0.43 2.08) | 0.90 |
| ALU 247 ≥ 21 & ALU 115 ≥ 41pg/ µL  ALU 247 < 21 & ALU 115 < 41 pg/ µL | 37  49 | 91.0  70.4 | 1.00  3.60 (1.03 12.53) | 0.03 |  | | | | |
| Integrity < 0.5  ≥ 0.5 | 41  45 | 81.9  75.8 | 1.00  1.26 (0.48 3.32) | 0.64 | < 0.48  ≥ 0.48 | 38  48 | 77.7  58.0 | 2.30 (0.96 5.52) | 0.05 |
